# User-Centered App Adaptation of a Low-Intensity E-Mental Health Intervention for Syrian Refugees

**DOI:** 10.3389/fpsyt.2018.00663

**Published:** 2019-01-25

**Authors:** Sebastian Burchert, Mohammed Salem Alkneme, Martha Bird, Kenneth Carswell, Pim Cuijpers, Pernille Hansen, Eva Heim, Melissa Harper Shehadeh, Marit Sijbrandij, Edith van't Hof, Christine Knaevelsrud

**Affiliations:** ^1^Division of Clinical-Psychological Intervention, Department of Education and Psychology, Freie Universität Berlin, Berlin, Germany; ^2^International Federation of Red Cross and Red Crescent Societies Reference Centre for Psychosocial Support, Copenhagen, Denmark; ^3^Department of Mental Health and Substance Abuse, World Health Organization, Geneva, Switzerland; ^4^Department of Clinical, Neuro- and Developmental Psychology, VU University Amsterdam, Amsterdam, Netherlands; ^5^Division of Psychopathology and Clinical Intervention, Department of Psychology, University of Zurich, Zurich, Switzerland

**Keywords:** e-mental health, mobile mental health, refugees, Syrian, global mental health, user centered design, psychosocial support

## Abstract

**Introduction:** The aim of this study is to describe the initial stages of the iterative and user-centered mobile mental health adaptation process of Step-by-Step (SbS), a modularized and originally web-based e-mental health intervention developed by the World Health Organization (WHO). Given the great need for improving the responsiveness and accessibility of health systems in host countries, the EU-funded STRENGTHS consortium studies the adaptation, implementation and scaling-up of SbS for Syrian refugees in Germany, Sweden and Egypt. Using early prototyping, usability testing and identification of barriers to implementation, the study demonstrates a user-centered process of contextual adaptation to the needs and expectations of Syrian refugees.

**Materials and Methods:** *N* = 128 adult Syrian refugees residing in Germany, Sweden and Egypt took part in qualitative assessments. Access, usage, and potential barriers regarding information and communication technologies (ICTs) were assessed in free list interviews. Interactive prototypes of the app were presented in key informant interviews and evaluated on usability, user experience and dissemination strategies. Focus groups were conducted to verify the results. The interview protocols were analyzed using inductive and deductive thematic analysis.

**Results:** The use of digital technologies was found to be widespread among Syrian refugees. Technical literacy and problems with accessing the internet were common barriers. The majority of the respondents reacted positively to the presented app prototypes, stressing the potential health impact of the intervention (*n* = 28; 78%), its flexibility and customizability (*n* = 19; 53%) as well as the easy learnability of the app (*n* = 12; 33%). Aesthetic components (*n* = 12; 33%) and the overall length and pace of the intervention sessions (*n* = 9; 25%) were criticized in regard to their negative impact on user motivation. Acceptability, credibility, and technical requirements were identified as main barriers to implementation.

**Discussion:** The study provided valuable guidance for adapting the app version of SbS and for mobile mental health adaptation in general. The findings underline the value of contextual adaptation with a focus on usability, user experience, and context specific dissemination strategies. Related factors such as access, acceptability and adherence have major implications for scaling-up digital interventions.

## Introduction

Seven years after the start of the armed conflict in Syria, Syrians are still the largest refugee population worldwide. With more than six million internally displaced in Syria and more than 5 million who have fled Syria, the humanitarian impact of the war is profound and far-reaching at the individual as well as at the global level (1). As a consequence of the ongoing crisis, Syrian refugees are confronted with numerous sources of psychological distress caused by loss, grief and trauma as well as by post-migration stressors such as perceived discrimination, concerns about the safety of family members in Syria or the host country and social as well as economic strain (2, 3). The risk of adverse mental health consequences in refugee populations affected by severe distress is well documented (2, 4, 5). Post-migration factors are increasingly being recognized as crucial in determining the actual long-term risk of developing psychological symptoms in refugees (3, 6, 7). This includes limited access to local health systems, which is often more difficult for refugees due to a variety of barriers at the individual level (e.g., mental health literacy or fear of stigmatization) (8) and the structural level (e.g., a lack of trained staff, the language barrier or legal restrictions) (9, 10). Low use of mental health care services is a major reason for the chronic nature of PTSD and other mental health issues in refugees (11, 12). Improving the responsiveness and accessibility of local mental health systems through the identification, implementation and scaling-up of contextually appropriate preventive measures as well as low-threshold interventions for refugees therefore is a major challenge in the public health field (2, 9).

### The STRENGTHS Project

The EU Horizon2020 STRENGTHS (**S**yrian **RE**fu**G**ees Me**NT**al Heal**TH** Care **S**ystems) program is a joint effort by academic and research institutions from Europe, international organizations and humanitarian organizations to improve the responsiveness of mental health systems for Syrian refugees in Europe and key countries in the Middle East and Northern Africa (13). STRENGTHS builds upon the growing evidence-base for task-shifting, trans-diagnostic treatment approaches and culturally adapted interventions for common mental disorders in populations affected by adversity. One part of STRENGTHS is to investigate the potential of an e-mental health intervention (Step-by-Step) as a strategy for increasing access to treatment in two high-income European countries (Germany and Sweden) as well as one lower-middle-income country in Northern Africa (Egypt). This paper describes the adaptation of Step-by-Step for Syrian refugees.

### E-Mental Health for Refugees

E-mental health is defined as “the use of information and communication technology (ICT)—in particular the many technologies related to the internet—when these technologies are used to support and improve mental health conditions and mental health care, including care for people with substance use and comorbid disorders.” (p. 1) (14). Depending on delivery method or problem being addressed, e-mental health interventions may be as effective as respective face-to-face versions (15), while reducing the impact of common internal barriers and external barriers to accessing care (16, 17). With the global spread of smartphones and the corresponding replacement of computers as the main access point to internet-based services, e-mental health tools are increasingly being developed for mobile computing and communication technologies which has led to the diversion of mobile health (mHealth) and mobile mental health as sub forms of e-mental health (18). The evidence-base for mobile mental health apps is still very limited which is mainly due to a lack of randomized-controlled trials (RCTs) (19, 20), but findings for acceptability of such approaches are strong. Smartphones are of immense importance for current refugees (21) and there are promising initial findings on the acceptability of mHealth for those affected by war and disaster (22).

Syrian refugees in particular are being described as “the most tech-savvy population of migrants in history” (p. 22) (23). Consequently, the potential of making basic mental health information and skills available at a large scale through smartphones requires further exploration. However, studies on the use of information and communications technologies (ICTs) shed light on a number of barriers that need to be taken into account when developing mobile solutions for Syrian refugees. Among these are the limited access to smartphones in parts of the population (e.g., elderly people or women) (24), technical limitations (e.g., limited or unstable access to mobile internet), financial limitations (e.g., the cost of smartphones and SIM cards) but also important literacy limitations (including technology literacy). All of these aspects shape the ways refugees use their smartphones, which is why it is recommended to exercise caution in assuming that Syrians have the same needs and concerns in regard to apps that would apply for Western populations (23, 25). Consequently, the development of app-based interventions requires a contextually sensitive approach.

### Contextual Adaptation

Cultural adaptation (26) has been recognized as a factor that potentially increases the effectiveness of behavioral health interventions (27, 28). Recent guidelines on the implementation of culturally sensitive interventions for refugees describe cultural adaptation in terms of a broad approach to contextual adaptation, taking into account not only “culture,” but also broader access- and acceptability-related factors such as structural barriers that hinder intervention adherence (29). Consequently, contextual adaptation does not only apply to the content of an intervention (e.g., language and key topics), but also to the needs and expectations of potential users in regard to the digital medium itself, through which the intervention is offered. These needs and expectations are, in turn, shaped not only by the refugees (e.g., demographics, flight experiences) themselves, but also by their surroundings or context (e.g., living situation, host community attitudes toward refugees, levels of support provided by the host community). In the case of mobile health interventions, a key concept in this context is usability (30). Usability dimensions can have a significant effect on the acceptance and adoption of e-mental health programs (31) and are therefore highly relevant for mobile mental health programs that often suffer from low user engagement (i.e., how actively people are using the program) and low user retention rates (i.e., the percentage of users remaining after a given period of time) (32–34). Self-guided digital interventions in particular heavily rely on user engagement as they cannot build compliance and adherence on the basis of a client-practitioner relationship. High dropout rates and irregular patterns of use endanger the statistical power and the validity of the results in e-mental health trials as well as the final utility of the intervention at population level post testing in RCTs. Currently, only a few examples of systematic usability testing as an approach to optimizing the design, user engagement and relevance of mobile mental health programs exist (35–37). Following recommendations by the WHO mHealth Technical Evidence and Review Group (38), STRENGTHS aims to add to this body of research by documenting iterative user engagement at the formative research phase of the project.

The Integrate, Design, Assess, and Share (IDEAS) framework by Mummah et al. (39) is currently the most comprehensive set of published guidelines for an iterative eHealth (including e-mental health) development and evaluation process, emphasizing the importance of evidence based implementation strategies, elements of design thinking, user-centered design and early prototyping. The framework encourages user-centered solutions that are based on an assessment of the actual needs and living conditions of the target population and is therefore well-suited for the contextual adaptation of behavioral interventions for refugee populations. The IDEAS framework suggests 10 phases of intervention development: (1) empathize with target users, (2) specify target behavior, (3) ground in behavioral theory, (4) ideate creative implementation strategies, (5) prototype potential products, (6) gather user feedback, (7) build a minimum viable product (MVP, i.e., the first fully-functioning version of the program that includes all core features), (8) pilot test, (9) evaluate efficacy, and (10) share widely. The phases of the IDEAS framework are intended to be recurring and interwoven. Phase (1) in particular, represents user engagement at all stages of the development process. Within STRENGTHS, the phases (2) and (3) are covered by working with the existing e-mental health intervention concept “Step-by-Step” (SbS) as a basis for further mobile mental health adaptation.

### Step-by-Step

Step-by-Step (SbS) is an e-mental health intervention developed by the WHO for depression. SbS was originally conceptualized as an online self-help version of WHOs evidence-based Problem Management Plus (PM+) program (40–42). The intervention is part of a group of WHO evidence based psychological interventions that all share task-shifting and a strong focus on potential scalability as basic principles in reducing psychological distress and improving functioning in communities affected by adversity. The process of developing SbS and its content are described in more detail in a paper by Carswell et al. (43). In order to provide high adaptability, SbS consists of three core components: the content, the guidance model (e.g., from a human helper) and the delivery system (e.g., web or app). Each of these components can be adjusted, extended, and combined to create versions of SbS with a strong focus on acceptability, usability and feasibility to respond to diverse implementation contexts. SbS is modularized and rooted in evidence-based cognitive behavioral therapy (CBT) techniques such as behavioral activation, psychoeducation, stress management, increasing social support and relapse prevention. The SbS content comprises of sessions that tell a story through illustrated educative narratives and interactive exercises presented by a fictional main character and a fictional health professional (43). At the guidance level, SbS is a self-help intervention that can be offered with weekly minimal guidance in the form of contact with a trained and supervised non-specialist (called an “e-helper”), or with contact-on-demand or no guidance. At the delivery system level, SbS is suitable for a wide range of mediums to increase access to diverse user groups (e.g., illiterate users, or users without access to the internet), including websites, apps, audio, video or books (43).

The first version of SbS was developed, culturally adapted and piloted for use by Syrian, Lebanese and Palestinian populations in Lebanon (44). It addresses depression, is web-based, presents content in the form of Levantine Arabic texts (i.e., a broad Arabic dialect spoken by Syrians, Lebanese and Palestinians) with illustrations and provides minimal guidance through weekly e-helper contact. This paper builds on the earlier work of WHO and focuses on the phases (4–6) of the IDEAS framework. It reports on the early formative stages that were conducted to create and user-test initial prototypes of a second version of SbS that is optimized for self-guided use on smartphones and for a contact-on-demand guidance model through messaging (i.e., to provide assistance with questions on the program as well as technical support).

## Materials and Methods

### Data Collection

The data for this study was collected as part of the Rapid Qualitative Assessment (RQA) phase of the STRENGTHS project under the lead of the International Federation of Red Cross and Red Crescent Societies (IFRC) Reference Centre for Psychosocial Support based at Danish Red Cross. The aim of the RQA was to gain quick input from the target population using pragmatic data collection methods. Across all eight sites of the project, a shared adaptation protocol was developed on the basis of established methodologies developed by the WHO (45) and the Applied Mental Health Research (AMHR) Group at Johns Hopkins University (46). Based on module one of the AMHR Development, Implementation, Monitoring, and Evaluation (DIME) manual, three phases of qualitative research were conducted with adult Syrian refugees (18+ years) living in Germany, Sweden and Egypt. The three phases utilized different data collection methods: free list interviews in phase one, key informant interviews in phase two, and focus group discussions in phase three. Throughout all phases, data were collected by Arabic native speakers who received separate training sessions for the respective assessment method prior to each phase of data collection. The interviewer teams in Germany and Sweden were composed of Syrians while the team in Egypt consisted of Egyptians. In an iterative process of early prototype development, the user input collected in each phase was integrated into an initial prototype (presented in phase two) and a slightly updated version of the app prototype (presented in phase three).

Over all three phases and countries, a total of *N* = 128 adult Syrian refugees participated. To address privacy concerns, no audio recordings were used, neither in interviews nor focus groups. Instead, interviewers were trained to work in pairs, one asking the questions and the other creating a written record. After each interview, the two interviewers discussed and—where necessary—added to the protocol. This pragmatic approach was chosen in accordance with the DIME methodology in order to reduce response bias, time investment for transcription and the risk of violating confidentiality, as well as to enhance interviewer fidelity to the interviewing methods (46). Participants were remunerated financially for their time.

### Phase 1: Free List Interviewing

The first assessment phase focused on the use of ICTs and potential problems associated with this among Syrian refugees residing in Egypt, Germany and Sweden. At this stage, free list interviews were conducted. This method uses standardized questions in a highly structured interview format that generates answers in the form of a list (46). The aim of free list interviews is to gain a quick overview of a relevant topic.

#### Participants

In each country, free list interviewees were recruited through Maximum Variation Sampling (MVS). MVS is a form of purposive sampling that aims at achieving a heterogeneous sample on a pre-selected number of key characteristics of the population. The free list interviews were conducted between July and August 2017. Recruitment in each country was facilitated through a combination of advertising and directly approaching Syrians within communities and NGO networks, as well as through snowballing. In order to include different perspectives on ICTs among Syrian refugees, we used MVS in order to approximate equal quotas with regard to gender, age, and level of education. Apart from being 18 years or older and the MVS criteria, there were no other inclusion or exclusion criteria. In particular, there was no screening for symptoms of psychological distress. While there is no gold-standard for sample sizes in qualitative research, *n* = 20 interviews per country were considered as sufficient to gain a solid overview of the most important themes (47). The mean age of participants was 33.0 years (*SD* = 11.0). On average, they lived in their respective host countries for 3.1 years (*SD* = 1.8). 46.7% had a university level education background, 51.7% had a secondary school background and 1.7% had a primary school background.

#### Topics

Interviewers were instructed not to lead interviewees during the free list interview phase. To elicit more generic statements and to further protect the interviewees' privacy, all questions were asked in regard to the community (i.e., Syrian refugees residing in the respective country) and not in regard to the individual being interviewed. Participants were therefore encouraged by interviewers to think of responses that describe what is typical for the group of Syrian refugees as a whole. Among others, the following questions were asked (further questions focused on common problems and functioning that are not subject of this paper):

1. What digital technologies do Syrian refugees frequently use?
2. What problems or difficulties can occur when Syrian men/women use digital technologies like smartphones, computers, apps or the internet?
3. What could be done to overcome these problems or difficulties?

For each point mentioned, participants were asked to provide a short description. Participants were also asked to recommend key persons in their communities that could be approached as key informants for phase 2 of the study.

### Phase 2: Key Informant Interviewing

**Figure 1:**
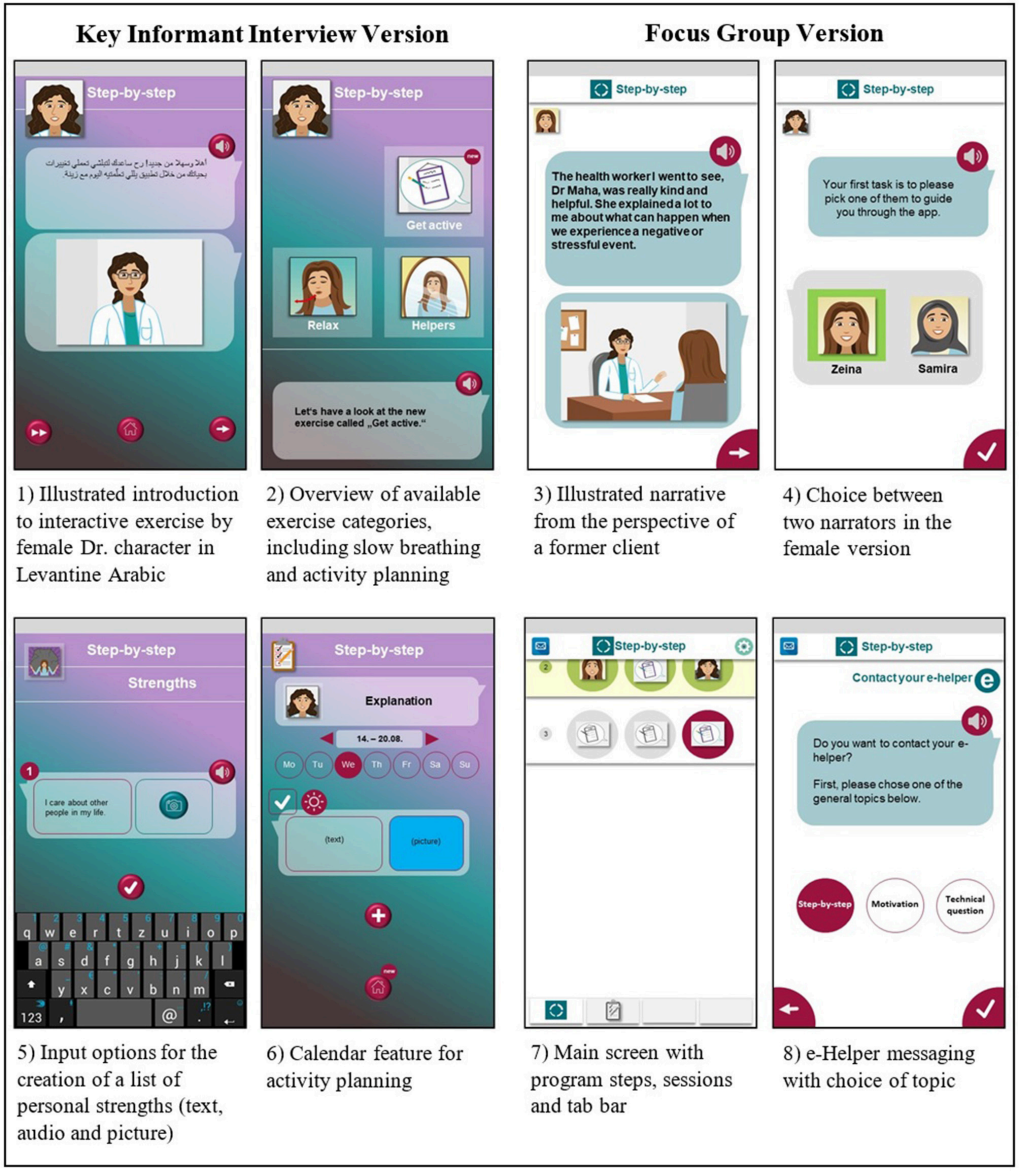
Selected prototype screens (text on screen 3 and illustrations depicting Step-by-Step characters and icons are adapted and reproduced with permission from WHO).

The IDEAS framework recommends conducting early usability testing, where participants are observed while using the system and asked to “think aloud.” With this method, relevant (implicit and explicit) information on users' interest is gained, and specific aspects in the system that potentially foster or hinder its use are easily identified. In addition, early usability testing based on prototypes may provide new perspectives on the app that can lead to improvement or solutions not yet considered. Using the online prototyping software InVision by InVisionApp Inc. (2017), an interactive prototype of smartphone adapted SbS was created and presented to key informants recruited within Syrian refugee communities in each country. The prototype included the onboarding and introduction session of SbS in which users receive information on the intervention, answer screening questionnaires and create an account. As part of the introduction, users were also introduced to a slow-breathing exercise for relaxation. The prototype further included session 1 of SbS that focusses on behavioral activation through psychoeducation and introduces the planning of enjoyable activities (43, 44). For the interview, mobile devices were handed to participants and they were asked to interact with the SbS app prototype specifically developed for this purpose. Figure 1 presents a selection of key screens included in this prototype.

#### Participants

Twelve key informants were interviewed per country, resulting in a total of *N* = 36. At this stage, potential interviewees were selected based on their role as a key person within the community and their knowledge about the problems that Syrian refugees commonly face in the host countries. These participants were identified based on previous participant's recommendations and through local organizations. The key informant interviews were conducted between September and October 2017. In this phase, no attempt was made to get a variety in respondents in terms of age or education level. However, an equal number of men and women were included. The mean age of the key informants was 33.8 years (*SD* = 10.9). On average, they lived in their respective host countries for 3.5 years (*SD* = 1.3). 83.3% had a university level education background while the rest of the sample had a secondary school background.

#### Topics

Interviews with key informants while they were using the prototype app focused on their initial impressions and feedback. Respondents were first presented with a one-page information sheet providing general information about the SbS program. Afterwards, the interactive app prototype was presented and participants had the opportunity to test it.

While going through the information sheet and the app, participants were invited to freely express their thoughts, comments and feedback on what they saw, thought or experienced. These were written down by the interviewers. Subsequently, key informants were asked the following standardized questions regarding the intervention prototype:

1. Do you think that this app can be helpful for Syrian refugees here in [country] who experience sadness or distress?
2. What do you think is good about the app?
3. What do you think is not so good or bad about the app?
4. What could be done to improve the app?

In addition, interviewees were asked about potential barriers and facilitators for the use of the app:

5. What do you think might stop or prevent Syrian refugees here in [country] from using the app?
6. What do you think could be done to motivate Syrian refugees here in [country] to use the app?

### Phase 3: Focus Group Discussions

Following the key informant interviews, the initial prototype was revised and a slightly adjusted second version (see Figure 1) was presented to participants in two focus groups per country, one with male and one with female participants, respectively. Focus groups were conducted with the following aims: (1) triangulation (i.e., using a different assessment method to verify previous findings) and (2) gathering feedback on changes that were made to the first prototype.

#### Participants

At this stage, the phase two recruitment procedures were repeated. A total of *N* = 32 persons participated in Germany (male: *n* = 5, female: *n* = 6), Sweden (male: *n* = 5, female: *n* = 4), and Egypt (male: *n* = 6, female: *n* = 6). The focus groups were conducted between October and December 2017. Apart from gender, no demographic characteristics were assessed for focus group participants.

#### Topics

Participants received the same information sheet that was used in phase two and had time to go through the prototype on a smartphone. After that, the group discussed the same questions that were asked in the key informant interviews while the interviewers created a written record. In order to gain feedback on the planned contact-on-demand feature, focus group participants received a description of the feature stating that users of the app can contact trained e-helpers (i.e., university graduates with a background in psychology) to ask questions regarding: (a) The SbS program, (b) issues around motivation and (c) technical issues. It was further stated that messages can be sent through a messaging system in the app and that e-helpers will reply within 48 h. Based on this information and a section in the interactive prototype demonstrating the feature, participants were then asked to give their feedback on the concept and to discuss whether e-helpers should have access to user input (i.e., texts, picture or audio input as part of the interactive exercises).

### Data Analysis

Prior to data analysis, all interview transcripts were translated into English. Data was coded using the NVivo version 11 qualitative data analysis software by QSR International Pty Ltd. (2017). The data was analyzed by two independent researchers. One worked with the Arabic original transcripts (second author) and one with the translated transcripts (first author). Ambiguities and deviating results of the qualitative data analysis were discussed and resolved in consultation between both data analysts.

#### Free List Interview Data

All participant responses to the respective free list interview questions were listed and coded without a pre-existing coding framework. Multiple responses that reported the same information were grouped and the number of participants mentioning that aspect was noted. Responses that were different in wording but similar in meaning were combined and a shared wording was found. Through this inductive approach, a common coding framework for the free list interviews in all three countries was developed. This resulted in a list of responses for each free list question and quantitative data on the total number of interviewees reporting each entry (see **Table 2**). The relative frequency of the responses can be interpreted as an indicator of the importance of the item (46).

#### Key Informant Interview Data

The key informant interview protocols were analyzed using a combination of inductive and deductive thematic analysis (48). A pre-existing theoretical framework for usability testing, the Health IT Usability Evaluation Model (Health-ITUEM), was chosen as the basis for the coding frame used in the deductive analysis (49). The Health-ITUEM is a comprehensive usability evaluation model on the basis of ISO 9241-11 (30) and the Technology Acceptance Model (TAM) by Davis (50). The original set of usability dimensions was further adapted by Househ et al. (51) with the major addition of subjective health impact as a usability dimension. In accordance with recent publications on the factor structure of questionnaire items based on the Health-ITUEM, the dimensions were categorized and assigned to the following higher order themes: Impact, perceived usefulness, perceived ease of use and user control (52). As suggested by Brown et al. (49), each usability dimension was broken down into positive (+) and negative codes (–). No neutral codes were used (51), instead, a new code for suggestions (s) was included as interviewees were asked for ways of improving the prototype. Table 1 provides an overview of the usability dimensions used for deductive data analysis in this study. In order to further explore the data, responses that were not covered by the Health-ITUEM were coded and checked for themes in an inductive manner. Using the same approach as described above for the free list interviews, the responses that participants gave in regard to potential barriers and facilitators to using the app were analyzed, aggregated and listed (see **Table 5**).

**Table 1:** Definitions of the Health-ITUEM usability dimensions used for deductive data analysis (adapted based on 49, 51, and 52).

**Usability dimensions**	**Definitions**	**Examples (coding)**
**IMPACT**		
Health impact	Expected impacts of the Step-by-Step app on the mental health of Syrian refugees.	G: “It will indeed help people, especially those with mental problems because those people are really looking for help.” (+)
Information needs	The extent to which information content meets user's needs.	S: “Sometimes you don't know what is going on with you, but with such an app you can get an idea.” (+) S: “There are a lot of repeated information and ideas.” (–)
Other outcomes	Other system-specific outcomes representing higher levels of expectations.	S: “If I really need something like this I could browse the internet to find out the solution to my problem or I could visit a psychologist.” (–)
**PERCEIVED USEFULNESS**		
Performance speed	Temporal efficiency when completing tasks within the app (e.g., learning from a narrative or practicing a technique).	S: “It should be short, clear, concentrated and up-to-date and shouldn't need a long time to use or it will be boring.” (s)
Flexibility and customizability	Providing alternative ways for accomplishing tasks, which allows different users to operate the system as preferred.	G: “I can use it at home anytime I want.” (+) E: “The availability of audio for those who cannot read.” (+)
**PERCEIVED EASE OF USE**		
Learnability	First-time users are easily able to understand and operate the Step-by-Step app.	S: “It is easy to use, simple language and the vocabularies are not difficult everyone can understand them.”
Competency	Users express confidence in their ability to use the Step-by-Step app.	(This theme did not occur in the data.)
Memorability	Users can remember easily how to perform tasks through the Step-by-Step app after not using it for a while.	(This theme did not occur in the data.)
**USER CONTROL**		
Error prevention	The Step-by-Step app offers error management, such as error messages as feedback, error correction through undo function, or error prevention, such as instructions or reminders, to assist users performing tasks.	(This theme did not occur in the data.)

*Responses were coded as either positive (+), negative (–) or suggestion (s), E, comment from Egypt; G, comment from Germany; S, comment from Sweden*.

#### Focus Group Data

Focus group data was analyzed at the group and not at the individual level. Themes that were mentioned or discussed within a group were coded according to the complete coding scheme that was created in the process of analysing the key informant data—including themes that were found in the inductive analysis. The results are reported separately to complement or contrast the key informant interview findings.

## Results of Free List Interviews, Key Informant Interviews and Focus Groups

### Information and Communication Technologies (ICTs)

The use of digital technologies was found to be widespread among Syrian refugees in Germany, Sweden and Egypt. Table 2 gives an overview of the responses that free list interviewees gave when asked about popular ICTs, common problems that occur when Syrian refugees use ICTs and suggested means of improving the use of ICTs. All of the main themes displayed in Table 2 were identified after analyzing a maximum of 11 out of the 20 interviews per country.

**Table 2:** Results on the use of Information and Communication Technologies (ICTs) found in free list interviews in Germany, Sweden and Egypt (each *N* = 20).

		Number (n) and percentage of coding references per country					
		Germany		Sweden		Egypt	
**FREQUENTLY USED ICTs**						
**Devices**						
1	Smartphones	14	(70%)	10	(50%)	9	(45%)
2	Laptops	6	(30%)	1	(5%)	2	(10%)
**Software**						
1	WhatsApp	6	(30%)	13	(65%)	20	(100%)
2	Facebook	14	(70%)	19	(95%)	16	(80%)
3	Viber	–	–	8	(40%)	14	(70%)
4	IMO messenger	–	–	2	(10%)	12	(60%)
5	YouTube	7	(35%)	1	(5%)	8	(40%)
6	Banking apps	–	–	6	(35%)	–	–
7	Official government service apps	–	–	9	(45%)	–	–
**FREQUENT PROBLEMS WITH ICTs**						
1	High costs of mobile internet	–	–	1	(5%)	14	(70%)
2	No access to landlines	–	–	–	–	10	(50%)
3	Low technical literacy	5	(25%)	10	(50%)	8	(40%)
4	Bad mobile internet access/coverage	7	(35%)	–	–	6	(30%)
5	High costs of smartphones	–	–	–	–	5	(25%)
6	Language barrier	8	(40%)	7	(35%)	5	(25%)
**MEANS TO IMPROVE THE USE OF ICTs**						
1	Increasing the technical literacy	6	(30%)	7	(35%)	9	(45%)
2	Providing easier internet access	4	(20%)	1	(5%)	8	(40%)
3	Reducing the costs of mobile internet	–	–	1	(5%)	5	(25%)
4	Providing versions in Arabic	7	(35%)	3	(15%)	2	(10%)

*Participants were asked to respond for the Syrian community as a whole and not on the basis of individual preferences or problems*.

#### Use of ICTs

Social media and digital communication technologies were mentioned most frequently in all three countries. Facebook was found to be the most widespread communication platform and was also often mentioned as a primary source of news and information. Smartphones were described as the main access technology to all kinds of information, communication and entertainment services on the internet. While smartphones were described as universal devices, laptops were rarely mentioned and described as devices that were mainly used for education purposes but also as too expensive for most Syrians. Only in Sweden, Syrian refugees also reported using apps for activities that would otherwise require visits to administrative government offices or banks. These apps are used, e.g., in order to manage the job search with the employment office or to exchange information with the social security office. In addition, it was mentioned that financial transactions are often done via banking apps by Syrians in Sweden.

#### Problems With ICTs

In all three countries, respondents mentioned low technical literacy as well as limited language skills as hindering factors to using ICTs. In Germany, the most common problem reported was that many highly relevant webpages (e.g., government webpages) were only available in German or English. Technical literacy was the most common problem mentioned in Sweden. Respondents described the requirements as high and the potential consequences of mistakes as serious, as it was required to be able to use digital identification, app-based banking and to interact with a technically advanced administration. The interviewees stated that mistakes could cause loosing claims on jobs or living space as well as unwanted outcomes due to wrong privacy settings on social media accounts. The responses from Egypt were more related to financial issues. The most reported problem was the high cost of smartphones and mobile data packages that were described as not sufficient for standard usage. This problem was further exacerbated by Syrians reportedly not being allowed to apply for access to landlines at rented homes in Egypt.

#### Means to Improve ICT Use

Asked about potential solutions for problems with ICTs, participants in all three countries suggested measures to improve technical literacy (e.g., training courses or multimedia tutorials). In Sweden it was added that these courses should specifically focus on government services. The interviewees also suggested to supply Arabic language support for all important services and to make sure that privacy settings in digital services were clearer and well understood. In Egypt, it was suggested to make access to the internet easier and to reduce the costs of mobile data packages.

### Usability Dimensions

A number of usability themes in accordance with the Health-ITUEM were identified. Table 3 summarizes the data. Percentages in the text below are provided to illustrate usability sub-themes that at least two independent respondents or focus groups commented on. 27 out of the 36 key informant interviews provided 90.5% of the codes that were used in the final coding scheme.

**Table 3:** Overview of feedback on the app prototype gathered through key informant interviews (*N* = 36) and focus groups (*N* = 6) in Germany, Sweden and Egypt (combined).

	**Coding references per feedback type:**											
	**Number (n) and percentage**											
**Themes**	**Positive (+)**				**Negative (–)**				**Suggestion (s)**			
	**KI**		**FG**		**KI**		**FG**		**KI**		**FG**	
**IMPACT**^**a**^												
Health impact	28	78%	6	100%	5	14%	3	50%	11	31%	2	33%
Information needs	5	14%	3	50%	9	25%	1	17%	7	19%	-	-
Other outcomes	2	6%	2	33%	6	17	1	17%	10	28%	3	50%
**PERCEIVED USEFULNESS**^**a**^												
Performance speed	-	-	2	33%	9	25%	5	83%	8	22%	3	50%
Flexibility/Customizability	19	53%	5	83%	4	11%	2	33%	11	31%	4	67%
**PERCEIVED EASE OF USE**^**a**^												
Learnability	12	33%	4	67%	8	22%	4	67%	11	31%	-	-
**USER EXPERIENCE**^**b**^												
Perceived credibility	8	22%	3	50%	4	11%	1	17%	3	8%	1	17%
Anonymity	9	25%	4	67%	-	-	-	-	-	-	-	-
Motivation	14	39%	4	67%	10	28%	3	50%	10	28%	4	67%
Aesthetics	8	22%	4	67%	12	33%	4	67%	10	28%	2	33%
Cultural adaptation	13	36%	2	33	5	14%	4	67%	9	25%	1	17%

KI, key informant interviews; FG, focus groups.

a Themes based on the Health-ITUEM (dimensions without codes are omitted).

b *Additional themes generated through inductive data analysis*.

#### Health Impact

The majority of participants in key informant interviews (KI, *N* = 36) and focus groups (FG, *N* = 6) commented positively on the expected mental health impact of the SbS app when offered to Syrian refugees suffering from psychological distress. The respondents either generally considered the app to be useful (KI: *n* = 19; 53%; FG: *n* = 6; 100%) or further specified that it may be useful when dealing with stressors such as war memories or integration problems (KI: *n* = 10; 28%, FG: *n* = 2; 33%). Furthermore, respondents indicated that the app may improve access to psychological help for Syrians (KI: *n* = 9; 25%, FG: *n* = 2; 33%) and that it may help with feelings of loneliness, isolation, anxiety and depression as well as trauma-associated symptoms (KI: *n* = 6; 17%, FG: *n* = 3; 50%). The following key informant comment from Egypt illustrates the great need for easily accessible means to address psychological problems:

“*Syrians are in a big need of such an app, especially those who are newly coming to Egypt because they are totally in shock of the Egyptian community. Men and youth also need this app, they suffer from many psychological problems and they do not have a lot of time because of their work.”*

While the majority of the participants commented positively on the app's potential mental health impact, some participants in the key informant interviews expressed skepticism, stating that the app will not very likely help those who suffer from severe problems or have experienced potentially traumatic events (KI: *n* = 5; 14%). Very rarely, users commented that using the app may result in negative consequences such as feeling more isolated or depressed (KI: *n* = 1; 3%, FG: *n* = 1; 17%). Respondents provided various suggestions on how to improve the health impact of the app. One common suggestion was to provide contact with a real person, e.g., via chat or phone (KI: *n* = 7; 19%, FG: *n* = 2; 33%).

#### Information Needs

A number of comments in the key informant interviews referred to whether the information provided within the app prototype met the participants' information needs. Positive comments described the content and sequence of the sessions to be useful and relevant (KI: *n* = 5; 14%, FG: *n* = 3; 50%), while negative comments mainly focused on the content being too repetitive (KI: *n* = 7; 19%). Some participants made suggestions, e.g., to include links to external sources of information and support such as websites (KI: *n* = 4; 11%) and to provide additional information regarding local health systems (KI: *n* = 2; 6%).

#### Other Outcomes

Following the Health-ITUEM definition (49), the “other outcomes” category was coded whenever participants made references to non-phone app technology (i.e., books), non-mobile resources (i.e., therapists, parents, teachers, siblings), and other health related entities not directly related to the usability of mHealth. This was most often the case when participants expressed that the app cannot replace a psychologist or psychiatrist (KI: *n* = 6; 17%) or speaking to a real person in general (FG: *n* = 1; 17%). Consequently, some participants suggested implementing a referral system to ensure that severe cases see a therapist (KI: *n* = 5; 14%, FG: *n* = 2; 33%).

#### Performance Speed

This usability dimension is given if users are able to use the system efficiently and get to the features or information they want in a subjectively appropriate amount of time. Here, several participants commented on the general time requirement of going through a narrative session of the intervention. The main points of criticism were the length of the sessions due to the long text passages (KI: *n* = 6; 17%) and the resulting need to go through a large number of screens (KI: *n* = 2; 6%). Respondents suggested to reduce the overall amount of text (KI: *n* = 3; 8%), to provide an interface for quicker navigation through content screens (KI: *n* = 3; 8%) and to provide shorter sessions at a higher frequency (KI: *n* = 2; 6%). One interviewee from Germany said:

“*The program must be faster. I mean there should be more sessions every week, for example, a session every 3 days.”*

Based on this suggestion, the length and frequency of the prototype' s sessions where slightly adjusted, resulting in shorter but more frequent sessions in the focus group prototype. This was commented on positively in *n* = 2 of the focus groups (33%), but participants in the majority of focus groups (FG: *n* = 4; 67%) still perceived some parts to be overly lengthy, especially the introduction section of the app, and suggested further text shortening (FG: *n* = 3; 50%).

#### Flexibility and Customizability

Many participants regarded this usability dimension as a strength of the presented app prototype. Participants often commented positively on the option to play text as audio (KI: *n* = 8; 22%, FG: *n* = 2; 33%), the possibility of using the app regardless of time and location (KI: *n* = 6; 17%) and the possibility of recording audio as an alternative method to (written) text input (KI: *n* = 5; 14%, FG: *n* = 2; 33%). Other perceived strengths were the planned offline capability of the app (KI: *n* = 5; 14%) and the choice of narrators with different clothing styles (KI: *n* = 2; 6%, FG: *n* = 2; 33%). A key informant from Sweden stated:

“*The pictures are very useful and the idea of having many options and a girl with hijab and the other without is very nice.”*

Negative feedback in regard to the prototype's flexibility and customizability were rare and included the strictly linear nature of the sessions (KI: *n* = 2; 6%, FG: *n* = 1; 17%). The font size was commented to be too small especially for older users and not adjustable (KI: *n* = 2; 6%, FG: *n* = 1; 17%). As a consequence, a few participants suggested implementing different font size settings (KI: *n* = 3; 8%).

#### Learnability

Learnability is defined as the experienced ease of use and clarity when starting the app for the first time. One third of the participants commented positively on this dimension, mainly indicating that the prototype was generally easy to use (KI: *n* = 8; 22%, FG: *n* = 4; 67%) and that the language used was easy to understand (KI: *n* = 5; 14%, FG: *n* = 2; 33%). Problems with learnability were mostly related to using the app through the graphical user interface (GUI) (KI: *n* = 6; 17%). It was revised for the focus group prototype, in which—with one exception—no further difficulties with the GUI were identified. Very few interviewees indicated that they did not understand the features of the app such as the exercises or the purpose of the camera feature (KI: 2; 6%, FG: 2; 33%) and the choice between narrators (KI: *n* = 2; 6%, FG: *n* = 1; 17%). Asked about suggestions to improve the app, participants suggested providing a more practical explanation of the app's features and objectives (KI: *n* = 9; 25%) as well as improving the GUI to make navigation for less technically literate users easier (KI: *n* = 2; 6%).

#### Other Usability Dimensions

The remaining Health-ITUEM usability themes “competency,” “memorability” and “error prevention” were not found within the interview and focus group data.

### User Experience

Through inductive data analysis, a number of additional themes was identified. These included subjective accounts on how the participants felt regarding the app and were therefore subsumed under the global dimension of user experience (UX). UX is defined in ISO 9241-210 (53) as “A person's perceptions and responses that result from the use and/or anticipated use of a product, system or service.” (section 2.15). While usability themes represent the functional dimensions of using the app prototype, UX themes refer to the more subjective dimension of the tester feedback. Both aspects are closely interlinked with each other and with common barriers to using technologies. Table 4 provides an overview of these themes, including definitions and examples of responses taken from the interview protocols. All of the main user experience themes (e.g., aesthetics) were identified after 9 out of the 36 interviews and 91.4% of the codes were identified after 27 interviews.

**Table 4:** Definitions of user experience themes identified in the data through inductive analysis.

**UX dimensions**	**Definitions**	**Examples (coding)**
Perceived credibility	Users express that they trust the app or consider it a reliable source of help for Syrian refugees experiencing psychological distress.	E: “It's really obvious that the app contains privacy.” (+) S: “How can I trust it? Is this medical information accurate?” (–)
Anonymity	Feelings in regard to the anonymity of using an app instead of seeing a professional in person.	G: “In my opinion, this app could help people who feel a bit shy to be treated by a psychologist.” (+)
Motivation	Users express positive feelings toward the app such as being interested, excited or generally motivated to use the app.	E: “Anyone who will see the app will have curiosity to try it.” (+) E: “It is boring.” (–)
Aesthetics	User comments in regard to the visual design qualities of the Step-by-Step app prototype.	E: “The design is calming and relaxing.” (+) S: “The design is not attractive and not modern.” (–)
Cultural adaptation	Comments that refer to culturally adapted content of the app (i.e. narrative content, dialect and illustrations).	G: “The best thing is the spoken mother tongue.” (+) S: “There is real information and incidents all of us have been through.” (+)

*UX, user experience; responses were coded as either positive (+), negative (-) or suggestion (s); E, comment from Egypt; G, comment from Germany; S, comment from Sweden*.

#### Perceived Credibility

A large proportion of respondents indicated trust in the program by mentioning privacy or data security as particular strengths of the approach (KI: *n* = 8; 22%, FG: *n* = 2; 33%). An interviewee from Egypt said:

“*I have curiosity to know more, especially since I feel that the topic is handled with privacy and confidentiality.”*

Only very few participants expressed concern, stating that they did not consider the app a reliable source of information (KI: *n* = 4; 11%, FG: *n* = 1; 17%) or that they did not receive enough information at the beginning (KI: *n* = 2; 6%). Participants suggested to strengthen users' trust in the app by ensuring data protection (KI: *n* = 2; 6%, FG: *n* = 1; 17%), e.g., through a password system.

#### Anonymity

The option to receive a mental health program without having to see a professional face-to-face was commented on by several participants. The comments were exclusively positive and stated that users would potentially feel less shy, embarrassed, afraid, ashamed or generally more comfortable when using the app instead of seeing a professional (KI: *n* = 9; 25%, FG: *n* = 4; 67%). One participant from Sweden put this aspect into context:

“*Yes sure, especially in a country where we live in isolation mostly in winter, when we have our phones all the time in hand, it's nice to have some program like that home, easy going and that can help you when you're depressed while nobody else knows about that, this is a positive and interesting thing.”*

#### Motivation

Input on the motivation to use the app was a common theme for which both, positive and negative comments were given frequently. Positive statements expressed being interested, curious, impressed, motivated or generally liking the app (KI: *n* = 14; 39%, FG: *n* = 4; 67%). Negative comments almost universally referred to feeling bored or not interested due to the repetitive content and long text passages (KI: *n* = 10; 28%, FG: *n* = 3; 50%). Participants in the key informant interviews and focus groups proposed a number of improvements to increase user motivation, including: (1) motivating messages, (2) shorter and more frequent sessions, (3) entertaining elements such as quizzes, (4) reminders, and (5) regular feature updates in order to keep the app relevant beyond the initial 5 weeks.

#### Aesthetics

A number of participants commented positively on the visual quality of the app prototype, referring to either the colors (KI: *n* = 2; 6%) or the illustrations (KI: *n* = 4; 11%, FG: *n* = 1; 17%). However, negative comments on aesthetics were more common and mainly focused on not finding the colors suitable or attractive (KI: *n* = 6; 17%), the overall impression that the prototype did not meet the design quality of current apps (KI: *n* = 5; 14%) or considering the design of the app as childish (KI: *n* = 2; 6%). As a consequence, the colors and design were adjusted for the focus group prototype. In regard to the colors, the feedback was positive in *n* = 2 focus groups (33%) but negative in *n* = 4 focus groups (67%). Participants mainly suggested to improve the aesthetic qualities of the app by changing the design to be more modern (KI: *n* = 3; 8%), by picking more comfortable or joyful colors (KI: *n* = 3; 8%, FG: 2; 33%) or by using photos instead of illustrations (KI: *n* = 2; 6%, FG: *n* = 1; 17%).

#### Cultural Adaptation

More than one third of the key informants provided feedback that specifically addressed how they felt about the content that had originally been designed for Syrian, Lebanese and Palestinian populations in Lebanon. These comments almost exclusively referred to the texts that were presented in the spoken form of Levantine Arabic. It was indicated that this way of presenting information instead of in formal Arabic felt closer to the person, easier and more comfortable for users of the app (KI: *n* = 12; 33%, FG: *n* = 2; 33%). An interviewee from Egypt stated:

“*The language is good. It is very close to the Syrian accent, as if someone is speaking to you. One can understand this better because it is simple and not academic. Better than formal Arabic.”*

Another positive quality is related to how well respondents could identify with the content of the narratives (KI: *n* = 1; 3%, FG: *n* = 2; 33%). One participant in a focus group stated:

“*I am suffering from the same issues; this story is very similar to mine.”*

A few respondents indicated that they didn't like the spoken dialect (KI: *n* = 4; 11%, FG: *n* = 2; 33%) or the illustrations (KI: *n* = 4; 6%, FG: *n* = 1; 17%), indicating that the latter were either childish or not representative of Syrians. Consequently, it was suggested to offer more language options such as formal Arabic, Kurdish, Assyrian or Syriac as well as to consider using real photos or videos instead of illustrations. In some cases, participants commented on single terms that could trigger negative associations (KI: *n* = 3; 8%, FG: *n* = 1; 17%). The specific terms discussed in this regard were “patient” (
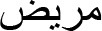
), “grief” (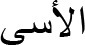) and “doctor” (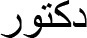).

#### Contact-On-Demand

The proposed feature was seen as not necessary in *n* = 2 focus groups (33%) and as useful in *n* = 3 focus groups (50%). Participants particularly pointed out that (1) e-helpers should not have access to user input within the app (FG: *n* = 5; 83%), (2) that only the e-helper (but no one else) should have access to such information (FG: *n* = 4; 66%), (3) that the 48-h response latency was too long (FG: *n* = 4; 66%), (4) that users should be allowed to decide what information they want to make available to e-helpers (FG: *n* = 3; 50%), and (5) that messages were preferred to calls (FG: *n* = 3; 50%).

### Barriers and Facilitators

Based on their first impression of the prototype, interviewees identified a number of potential barriers that might prevent Syrians from using the Step-by-Step app and suggested measures to facilitate the uptake of the program (see Table 5).

**Table 5:** Overview of barriers and facilitators to use of the Step-by-Step app gathered through key informant interviews (*N* = 36) and focus groups (*N* = 6) in Germany, Sweden and Egypt (combined).

**Number (n) and percentage of coding references**											
	**Barriers**	**KI**		**FG**			**Facilitators**	**KI**		**FG**	
1	Acceptability	16	44%	3	50%	1	Promotion (general)	14	38%	3	50%
2	Credibility	11	31%	5	83%	1(a)	Outreach	18	50%	5	83%
3	Technical requirements	8	22%	2	33%	1(b)	Social media	14	39%	6	100%
4	Technical literacy	6	17%	2	33%	1(c)	Personal recommendation	5	14%	4	67%
5	Too distressed	4	11%	1	17%		Tutorials or trainings	-	-	3	50%

*KI, key informant interviews; FG, focus groups*.

#### Barrier: Acceptability

Many of the key informants identified a lack of acceptance among Syrians as a major barrier. This was confirmed by participants in several focus groups and includes (1) the acceptance of having psychological problems, (2) the acceptance of the concept of psychological help itself, and (3) the acceptance of an app-based offer. An interviewee comment from Germany illustrates this:

“*The idea of going to a psychologist is a bit hard to accept for Syrians. Most people don't admit that a psychological illness is exactly the same as any other illness. I don't know if all will use this app for mental treatment.”*

#### Barrier: Credibility

Almost every third key informant and participants in all but one focus group mentioned a lack of trust in the app as a potential initial barrier to using it. The respondents assumed that this could either be due to worries about data protection or due to doubts whether the program will actually help. An interviewee from Germany stated:

“*Privacy? Is the data of the person really protected? Being unsure would prevent Syrians from using such an application.”*

#### Barrier: Technical Requirements

Interviewees and participants in focus groups further stated that a lack of smartphones or a lack of (reliable) internet connectivity would make it more difficult for Syrians to use the app. In addition, it was mentioned that this may especially affect older persons.

#### Barrier: Too Distressed

Another barrier mentioned by a couple of respondents was that some Syrians will be under too much psychological distress and therefore will likely not be willing or will not have the capacities to try the app. One interviewee comment from Egypt illustrates this point:

“*Generally there are no obstacles to the deployment of this app except for a minority of people who have really complicated circumstances.”*

#### Barrier: Technology Literacy

Finally, participants also identified limited familiarity with the technology as a barrier, especially among older and illiterate people as expressed by this interviewee from Sweden:

“*A very good idea but it won't be used by everyone, especially old people and people who can't read. So the idea will likely be spread among young people*.”

#### Facilitators: Promotion of the App

Asked about means to encourage Syrians to use the app, key informants and focus group participants exclusively mentioned different ways of promoting the app or the importance of making the app known in general. Three different approaches to the promotion of the app were identified in the data (see Table 5). The most commonly mentioned one was to conduct outreach in (1) community centers, (2) aid organizations, (3) government institutions, (4) language and integration classes, (5) public transportation, and (6) health care institutions. A second very common suggestion was to conduct social media campaigns and online advertising. As a third approach, a couple of respondents suggested focusing on personal recommendations by other Syrians who are using the app, either by training volunteers or by working with trusted key persons within the communities. Only in focus groups, the additional recommendation was given to provide tutorials or trainings on how to use the app.

## Discussion

This paper presents findings on the early stages of the iterative user-centered development and evaluation process of Step-by-Step (SbS)—a mobile mental health intervention being adapted for Syrian refugees with increased psychological distress and impaired functioning. The aim of the study was to conduct a rapid qualitative assessment on user needs and initial prototype feedback in three different country settings to inform decisions on the app's requirements profile (i.e., the list of required features and characteristics) for the subsequent software development and design process. To this end, three phases of data collection were conducted, starting with free list interviews on the use of information and communication technologies (ICTs) and followed by key informant interviews on main usability dimensions of an early interactive app prototype. The study was concluded with focus group discussions in which a slightly revised prototype version was presented. At all stages, barriers and facilitators to the use of ICTs in general (phase one) or SbS in particular (phases two and three) were assessed. The free list interviews provided an important overview of the context in which ICTs are used by Syrian refugees, while the key informant interviews and focus groups provided valuable feedback on the initial app prototype.

### Adaptation to Technical Literacy

It was found that the use of digital technologies was widespread among Syrian refugees in all three country settings and mostly occurred in the form of communication apps and social media that were accessed on mobile platforms by the majority of the target population. These findings are consistent with previous literature on ICT use among some refugee populations, underlining the omnipresent nature of mobile technologies (21) as well as the primary use of such technologies as communication tools (23). At the same time, low technical literacy was the most common barrier reported across all settings and in all three phases of data collection. As has been pointed out before (54), high usage of mobile phones among refugees does not necessarily mean a high level of overall technical literacy in this group. Instead, a significant proportion of Syrians may only be familiar with using smartphones within a very limited field of applications—namely as text or video messaging tools and as phones. In order to maximize the learnability of the app, essential features of the initial prototype were therefore designed in ways that were expected to feel familiar to users that have experience with messaging apps. For example, the narrative content screens (i.e., texts and illustrations) were presented in a format resembling prototypical messaging apps such as WhatsApp (see Figure 1). In addition, audio input via the phone's microphone and picture input via the phone's camera were added as alternatives to text input for all interactive exercises in order to increase the flexibility and customizability of the app. These input options are standard features in many messaging apps and were therefore expected to also be familiar to a large proportion of the intended users.

In the first version of the prototype, certain elements of the graphical user interface (intended to guide users through the program) were not understood by all respondents, e.g., a “new” icon to indicate new sections of the app. This was dropped for an interface design that was closer to the “step-by-step” metaphor of the intervention in the second version of the prototype. Sessions were now displayed on the main screen of the app as a sequence of steps. In order to further improve navigation, a tab bar was added. There was no indication of learnability issues around the interface of the revised version.

### Adaptation to Technical Barriers

Lack of (mobile) internet access was a major barrier and mentioned in all three phases of data collection. High costs as well as limited availability and coverage in certain regions were identified as the main causes of this barrier. Differences between the three countries became evident in the free list interviews as the issue was mainly thematized in Egypt, while participants in Sweden—a country with well-developed broadband coverage—did not mention mobile access and coverage at all. This supported initial work by WHO which had identified the need for any app to be usable in areas of poor internet. Given the shared aim of STRENGTHS and WHO to develop a mobile mental health solution with a high potential for scaling-up and robustness in diverse country settings, it was decided to design the app for less optimal conditions, including unstable and costly mobile connections. As many features of SbS as possible should therefore work offline after initial download, and use a minimum of mobile bandwidth (e.g., for data upload). The high cost of smartphones was mentioned quite frequently by free list interviewees in Egypt and was considered as another main barrier in conceptualizing the initial software requirements. It was concluded, that not all Syrians will be in possession of their own private devices. Instead, devices may be shared among family members. Previous research has shown that access to expensive communication devices tends to vary along age and gender lines. In a refugee camp context in Jordan, mobile phones were very common among younger Syrians (55), but also more often in the possession of male family members. Older women in particular often relied on their sons or grandsons when it came to the use of communication technologies (24). As shared devices could potentially lead to privacy issues due to the higher visibility of apps installed on mobile devices, it was decided to also provide a web-version of the program for use with standard mobile and desktop web-browsers. Instead of developing SbS as a native application, a hybrid approach to app development was chosen, resulting in an application for Android, iOS and web.

### Language Adaptation

A commonly mentioned barrier to using ICTs was language, as many technologies are only available in English or other local languages in the respective host countries, e.g., German or Swedish. Consequently, respondents suggested providing content in Arabic. This requirement was already met with the pre-existing SbS content in culturally adapted Levantine Arabic. While illiteracy itself was not mentioned in the free list interviews, there is indication of rising illiteracy rates in the younger generations of Syrian refugees who discontinued their education due to the war (54, 56). This suggests the importance of ensuring accessibility through an app. While the original web version of SbS addressed this to some degree by including videos of the narrative stories, the concept of the app extends this by making this feature easier to use through providing audio recordings of all texts in addition to the above mentioned audio input option. Respondents often pointed out that these features were of specific use to elderly or illiterate persons, underlining the importance of accessibility and barrier-free design (57).

Furthermore, respondents indicated that they found the prototype easy to use, not complicated and specifically pointed out the easy to understand language used in the texts. As this affects the potential applicability among diverse members of the target population, this usability dimension is closely related to the potential for scaling-up of a mobile mental health program (58). In addition to improving learnability, the language was also mentioned as a key positive aspect of the app's user experience. Participants mostly indicated that the texts in Levantine Arabic—which is similar to Syrian dialect—felt closer to the person. This theme often occurred in combination with expressions of interest, indicating that the cultural adaptation of the language not only improved ease-of-use but also engagement with the program. A lack of identification with narrative content has been identified as a barrier to adherence in previous e-mental health research and can occur when users feel that the information does not apply to them (59).

### Adaptations to Improve Acceptability

In regard to the potential health impact of SbS, the majority of the respondents in all three countries recognized the significance, importance and potential for improving Syrian refugee mental health care through a mobile mental health approach. Participants commented on the perceived value of the program's premise as well as on its potential positive effects for Syrians affected by distress. It is important to note that this feedback was gathered during and immediately after the very first encounter that respondents had with the prototype. It can therefore also be interpreted as an indicator of the immediate acceptability of the presented mobile mental health approach. This is further underlined by a large number of comments in which participants indicated being interested or curious about the app. This result is consistent with recent findings of a high interest and openness toward mobile mental health among Palestinians in the West Bank (60). On the other hand, not accepting the need for psychological aid or not accepting the mobile mental health approach itself were indicated as major barriers to the uptake of the program. Consequently, respondents were predominantly of the opinion that SbS can have a positive health impact, but only if it was accepted by the target group. This underlines the importance of addressing the acceptability of the intervention within the cultural context by considering factors such as health literacy, health beliefs (61) and mental health related stigma (62, 63). The positive early user feedback on this theme is promising as it indicates that the mobile mental health approach was well-received by many. However, some did not consider an app the appropriate medium for such a program. This facet of acceptability and its connections with potential user characteristics (e.g., symptom severity or health literacy) should be further investigated in order to clarify barriers to scalability and reach as well as appropriate measures to improve acceptability (e.g., avoiding certain terms, such as “patient”).

However, in line with the well-documented treatment gap experienced by refugees in their respective host countries (9, 10), participants mentioned common structural barriers and how the app might be an alternative way of accessing care, e.g., by being free of charge, available in Arabic and without geographical restrictions. In addition, respondents mentioned that users of the app may feel more comfortable with the approach as seeing a professional can be accompanied by negative feelings such as fear or shame. The anonymous nature of the program may therefore render it more acceptable and specifically attractive for those who would otherwise be reluctant to seek professional help due to fear of stigmatization or embarrassment (64). Both findings underline the potential of mobile mental health to provide an alternative to standard care for Syrians affected by individual as well as structural barriers to mental health care.

### Adaptations to Improve Credibility and Trust

Apart from acceptability, credibility was another very common barrier that may affect uptake. Here, respondents either indicated that potential users may not trust that the app will work (i.e., not having an effect on distress) or that they may not trust in the protection of their personal data. In this regard, several respondents specifically referred to the aspects of privacy and data protection. Trust is increasingly being recognized as an essential facet of system and software quality. It is one key aspect of UX and is defined in ISO 25010:2011 as the “degree to which a user or other stakeholder has confidence that a product or system will behave as intended” (section 4.1.3.2) (65). The standard further states that security is an essential contributor to trust. It is noteworthy that already at this early stage of user testing, participants in the key informant interviews and focus groups often pointed out the prototype's safety and credibility as positive aspects. While this indicates that the given information on data protection were successful in building trust, the findings also underline the importance of trust specifically within refugee populations that may struggle with trust in services due to potentially traumatic experiences in the past (66). The theme also occurred prominently when the e-helper system was discussed in the focus groups. Here, many respondents indicated that they would prefer that e-helpers did not see their inputs in the app at all or that the e-helpers should be the only persons to see this. Others suggested to give users control over what information they want to share with their e-helpers. This suggestion is in line with the principle of “privacy by default” (i.e., systems should be pre-configured for highest data protection, instead of expecting users to configure them accordingly). This principle was not only suggested by respondents in our study but has also recently become mandatory for systems collecting personal data in the European Union as part of the 2018 General Data Protection Regulation (67).

### Adaptation of the Guidance Model

One common misconception about e-mental health and mobile mental health is to consider them as replacements for existing sources of help while they are actually intended to provide alternatives. This can lead to higher expectations and may limit the acceptability of the approach. One common user comment in this regard was that the SbS app cannot replace a real psychotherapist. As this is not the aim of the approach, it is important to manage expectations as well as ensure clear interfaces with established structures within health care systems. STRENGTHS aims to not only provide scalable software but also identify barriers and facilitators to scaling-up within health care systems. To this end, user suggestions provided valuable insights. Key informants suggested to provide contact with professionals directly within the app. To learn more about what users expected from such a system, the planned contact-on-demand feature was included as part of the interactive prototype in the focus groups. Based on this, respondents either commented positively on the option or indicated that they did not consider it necessary to be able to contact e-helpers. Consequently, it was decided to keep contact optional. However, the suggested response latency of up to 48 h was universally considered as too long and needs to be reduced, e.g., through more efficient systems for e-helper management.

Furthermore, respondents suggested to integrate a referral system within the app to ensure that users with more severe symptoms can receive additional treatment or other forms of support by real persons. It was also indicated that too severe levels of distress may prevent Syrians from using the app. Here, a potential solution are stepped care models that often already include low-threshold digital programs like SbS for low severity cases and referral systems that give access to higher intensity face-to-face treatments for cases that are identified to be more severe or complex (68). Consequently, establishing gateways to existing health care structures should be considered from the early stages of software development on. Potential approaches to this can be (1) to include information (e.g., on the health care system in a country), (2) to provide contact information (e.g., to available emergency lines), or (3) to implement the program within existing mental health programs for refugees (e.g., offering it in treatment centers for war survivors).

### Adaptations to the Narrative Sections of the Program

The usability dimensions of performance speed and information needs received most of the negative feedback which pointed out critical areas for future improvement of the app's concept. In general, participants found the texts and sessions of the program too long (performance speed) and too repetitive (information needs), resulting in feeling bored or not interested in continuing to use the app. Adjustments are therefore crucial in order to ensure user engagement. This finding may indicate a general key area of adaptation that needs to be taken into account when transferring content from a web-based intervention to a smartphone format. While web-based interventions oftentimes work with longer text sections and session duration of up to 1 h, this format may not work as well on mobile devices due to smaller screens and different usage habits. Based on the clear user feedback, the content was restructured to fit into a format of overall shorter but more frequent sessions. A first attempt to shorten sections was presented in focus groups as part of the revised prototype. User feedback indicated that the changes may have improved but not solved the issue yet and that further adjustments to the overall length of sessions are required.

### Adaptations to Improve Aesthetics

Many respondents mentioned the aesthetic qualities of the prototype in direct association with the motivation to use the program. While the feedback was diverse, it became clear that many respondents had clear expectations on how a modern app should look like. As Bakker et al. (69) pointed out: “Building an enjoyable app with good graphic design and a slick, intuitive, and satisfying interface is necessary for an effective intervention.” (p. 13). Feedback on the revised prototype provided further insight into user expectations. On this basis, it was decided to involve a professional design company in the future development process.

## Limitations

A number of limitations have to be mentioned. As this was a rapid qualitative assessment approach following the guidelines of DIME module 1 (46), recruitment and data collection were conducted with a pragmatic focus. Especially working without audio recordings likely has affected data quality and data depth. The approach specifically limits possibilities to check data quality ex-post. This issue was addressed by providing training and clear guidelines to interviewers. Interviews were always conducted by two interviewers that compared their notes immediately after the interview in order to ensure the quality and completeness of the written records.

The study had the advantage of being able to recruit a larger sample than is usually feasible in early prototyping. Ex-post analyses on saturation indicated that the number of interviews was sufficient for the purpose of coding initial user feedback. During the inductive development of coding frameworks for ICT usage and user experience dimensions, 11 out of 20 free list interviews and 9 out of 36 key informant interviews were required to identify all of the themes. In addition, 27 out of 36 key informant interviews provided more than 90% of the codes. Due to the iterative nature of the adaptation process chosen for SbS, it was decided to first implement the conclusions drawn from this stage of qualitative interviews before recruiting additional interviewees. The generated coding frameworks will be re-used and where necessary extended in future iterations of user testing.

Throughout all phases of the study, an equal distribution of men and women was achieved. However, recruiting diverse respondents on other factors such as age or education was only attempted at the free list interviewing stage. While a heterogeneous age distribution was achieved, it was especially challenging to recruit participants with a lower education background. One reason for this may be that, before 2011, the Syrian education system was considered one of the most advanced in the Middle East with a high proportion of graduates with secondary education (70). In comparison with Syrians below the age of 18, the respondents in this study were therefore not as affected by the steep decline in school enrollment that started with the war (71). It is possible that the respondents in this study did not represent all layers of the Syrian refugee population. In the free list interviews, respondents still answered as representatives of the larger group instead of from a personal standpoint. Consequently, this approach may have introduced themes that were rather based on hearsay than on personal experience. While this cannot be ruled out, the results of the free list interviews were in line with previous research on the use of ICTs in refugee populations (21, 24, 55).

In addition, the interviewed refugees were not pre-screened for symptoms of distress. Consequently, the feedback does not necessarily originate from the exact target group of the app (i.e., Syrian refugees with increased distress and impaired functioning). While respondents were instructed to provide information from the communities' point of view or in regard to Syrians experiencing sadness or distress, the results may not necessarily reflect the impact of mental burden while using the app. Consequently, future user testing should also include clinical cases.

Other limitations are caused by the early nature of the prototype. Since respondents did not use the app for an extended period of time, feedback on usability dimensions such as health impact are based on first impression and not on an actual experienced effect. This may also have resulted in the absence of comments on the usability dimensions of competency, memorability, and error prevention. Future iterations of prototype testing should therefore enable users to try the app over a longer period of time in a natural environment. Since the prototyping software was used instead of providing a functioning version of an actual app, certain elements such as the interactive exercises could only be simulated at this stage. In order to receive actual feedback on a working prototype it will be necessary to provide access to a fully functioning version.

## Conclusions

Early formative research allows the immediate adaptation and improvement of app concepts and early prototypes for specific target populations such as Syrian refugees. It is a crucial first step toward pilot testing and subsequent randomized controlled trials and an important addition to the previous work on contextually adapting the SbS intervention (44). A usability and user experience focus is still rare in the field of e-mental health for refugees and has only recently started to emerge in the literature (72). To our knowledge, this is the first mobile mental health intervention for a refugee population that uses early prototyping and usability testing.

The qualitative assessments in this study provided valuable guidance for the mobile mental health adaptation of SbS and app development for refugee populations in general. The following recommendations can be derived from the results: mobile mental health apps should provide more sessions in shorter intervals than web-based interventions. Moreover, they should ensure intuitive user interfaces, provide a clear structure for less technical literate users and further improve motivation and engagement through interactivity. If contact-on-demand is used, it should happen with low response latencies.

User participation and usability evaluation will continue throughout the STRENGTHS project as part of the software development and process evaluation. Following the IDEAS framework, the next stage will be the creation of a minimum viable product (i.e., the first fully functioning version of the software). This version will be used in pilot RCTs to further evaluate its usability and feasibility in the study setting. Important topics such as the program's health impact and cost-effectiveness will be further assessed in definitive RCTs in the STRENGTHS project and by WHO in other RCTs. The qualitative results of the present study will inform the further process evaluation. While this study utilized rapid appraisal, other approaches such as questionnaires, in-depth interviews, “think out loud” sessions or user observation techniques will be used at later stages (73).

Given the valuable feedback that Syrian refugees provided in this study, user-informed approaches should find more application in the development of digital health projects for refugees and populations in low and middle income countries.

## Ethics Statement

This study was carried out in accordance with the recommendations of the ethics committee of the Department of Education and Psychology at Freie Universität Berlin. All subjects gave written informed consent in accordance with the Declaration of Helsinki. The protocol was approved by the ethics committee of the Department of Education and Psychology at Freie Universität Berlin.

## Author Contributions

SB, MB, PH, and CK designed and conducted the study with the support of MA, KC, PC, EH, MH, MS, and EvH. SB and MA analyzed the data and all authors contributed to the interpretation of the data. SB wrote the manuscript with support from MA, MB, KC, PC, PH, EH, MH, MS, EvH, and CK. All authors gave final approval of the version to be published. The authors alone are responsible for the views expressed in this article and they do not necessarily represent the views, decisions, or policies of the institutions with which they are affiliated.

### Conflict of Interest Statement

The authors declare that the research was conducted in the absence of any commercial or financial relationships that could be construed as a potential conflict of interest.
